# Investigation of niclosamide as a repurposing agent for skeletal muscle atrophy

**DOI:** 10.1371/journal.pone.0252135

**Published:** 2021-05-26

**Authors:** Hyun-Jun Kim, Ji-Hyung Lee, Seon-Wook Kim, Sang-Hoon Lee, Da-Woon Jung, Darren R. Williams

**Affiliations:** New Drug Targets Laboratory, School of Life Sciences, Gwangju Institute of Science and Technology, Gwangju, Jeollanam-do, Republic of Korea; University of Minnesota Medical School, UNITED STATES

## Abstract

Skeletal muscle atrophy is a feature of aging (termed sarcopenia) and various diseases, such as cancer and kidney failure. Effective drug treatment options for muscle atrophy are lacking. The tapeworm medication, niclosamide is being assessed for repurposing to treat numerous diseases, including end-stage cancer metastasis and hepatic steatosis. In this study, we investigated the potential of niclosamide as a repurposing drug for muscle atrophy. In a myotube atrophy model using the glucocorticoid, dexamethasone, niclosamide did not prevent the reduction in myotube diameter or the decreased expression of phosphorylated FOXO3a, which upregulates the ubiquitin-proteasome pathway of muscle catabolism. Treatment of normal myotubes with niclosamide did not activate mTOR, a major regulator of muscle protein synthesis, and increased the expression of atrogin-1, which is induced in catabolic states. Niclosamide treatment also inhibited myogenesis in muscle precursor cells, enhanced the expression of myoblast markers *Pax7* and *Myf5*, and downregulated the expression of differentiation markers *MyoD*, *MyoG* and *Myh2*. In an animal model of muscle atrophy, niclosamide did not improve muscle mass, grip strength or muscle fiber cross-sectional area. Muscle atrophy is also feature of cancer cachexia. IC_50_ analyses indicated that niclosamide was more cytotoxic for myoblasts than cancer cells. In addition, niclosamide did not suppress the induction of *iNOS*, a key mediator of atrophy, in an *in vitro* model of cancer cachexia and did not rescue myotube diameter. Overall, these results suggest that niclosamide may not be a suitable repurposing drug for glucocorticoid-induced skeletal muscle atrophy or cancer cachexia. Nevertheless, niclosamide may be employed as a compound to study mechanisms regulating myogenesis and catabolic pathways in skeletal muscle.

## Introduction

The global population has undergone a demographic shift towards the elderly. It is predicted that 202 million people will be over 80 years of age by 2030, and this will rise to 434 million by 2050 [1]. Consequently, medical research for this demographic has shifted from curing diseases to maintaining the ability to live independently [1]. A major risk factor impacting on independence is sarcopenia (the aging-related loss of skeletal muscle mass and function). Although there is debate about the definition of saropenia, it is estimated that this disease affects 5–13% of 60–70 year olds, and 11–50% of people over 80 years [2]. More than 400 million people are predicted to develop sarcopenia by the year 2040 due to population aging and the annual healthcare burden of sarcopenia is $19.12 billion in the United States [2,3]. There are currently no approved drugs for treating sarcopenia [4]. Medical interventions are based on nutritional supplementation and resistance training, which may suffer from non-compliance or the inability to exercise [5].

Skeletal muscle atrophy can also occur in advanced metastatic disease associated with certain types of cancer, such as pancreatic, breast or colorectal carcinoma (termed cancer cachexia). Patient quality of life and response to chemotherapeutics are significantly impacted by cancer cachexia [6]. A number of drug candidates are being assessed for cancer cachexia, including the xanthine oxidase inhibitor febuxostat, the antianginal metabolic agent, trimetazidine, and the nutritional supplement, salidroside [7–9].

Niclosamide (trade name Niclocide; abbreviated as Ni in this study) is a teniacide drug used to treat tapeworm infestations that was approved for human use over 60 years [10]. It is on the World Health Organization list of most effective and safe medicines, and a course of treatment costs around 0.25 US dollars [10]. Ni is being investigated for numerous drug repurposing applications, such end-stage metastasis, drug resistant *Staphylococcus aureus* infections, viral hemorrhagic fever, type 2 diabetes and hepatic steatosis [11–13]. In cells, Ni has been shown to inhibit the signal transducer and activator of transcription 3 (STAT3) and Wnt/β-catenin signaling pathways [14]. These pathways are also targets for sarcopenia drug development [15,16]. The Wnt pathway is known to crosstalk with Ras GTPase signaling, which has been linked to the development of sarcopenia [17]. STAT3-mediated signaling is also a target for cancer cachexia drug development [18].

In this study, we assessed the potential for repurposing Ni as a drug for skeletal muscle atrophy. The effect of Ni on cell viability was measured in skeletal muscle precursor cells (myoblasts) and differentiated myotubes. The therapeutic potential of Ni was tested in *in vitro* models of sarcopenia (dexamethasone-induced myotube atrophy), cancer cachexia (tumor necrosis factor-α treated myotubes) and myogenesis, in addition to an animal model of dexamethasone-induced skeletal muscle atrophy.

## Materials and methods

### Reagents

Ni was purchased from Sigma-Aldrich, NJ, USA (catalogue number N3510-50G) and Ni (ethanolamine salt) was purchased from Cayman chemical, MI, USA (catalogue number 17118-1G). Bovine insulin was purchased from Sigma-Aldrich (catalogue number 10516). Dexamethasone (abbreviated as Dex in this study) was purchased from Santa Cruz Biotechnology, TX, USA.

### Cell culture

HCT-116 (human colorectal carcinoma), A549 (human lung adenocarcinoma) and PANC1 (human pancreatic ductal carcinoma) were obtained from the Korean Cell Line Bank, (Republic of Korea). C2C12 (murine skeletal muscle myoblasts) were obtained from Koram Biotech. Corp, Republic of Korea. All cells were cultured with growth media (GM), consisting of Dulbecco’s Modified Eagle’s Medium (DMEM) supplemented with 10% fetal bovine serum (FBS), 1% penicillin and streptomycin (PenStrep).

Cell-based experiments were carried out within 2 months of thawing liquid nitrogen stocks. HCT-116, A549 and PANC1 cells used in this study were authenticated by short tandem repeat DNA fingerprinting at the Korean Cell Line Bank, using the AmpFLSTR identifiler PCR Amplification Kit (Applied Biosystems, CA, USA) and the 3730 DNA Analyzer GeneMapper ID v3.2 databases (Applied Biosystems). C2C12 cells were validated by their ability to differentiate into multinucleated myotubes. The cell lines were routinely assessed for Mycoplasma contamination at the Animal House Core Facility of the Gwangju Institute of Science and Technology, Gwangju, Republic of Korea, using the e-Myco Mycoplasma PCR Detection Kit (Intron Biotechnology, Republic of Korea). The C2C12 myoblasts used in this study were at passage number 16 to 23.

Differentiated skeletal muscle fibers (myotubes) were induced by treatment with differentiation media (DM: DMEM supplemented with 2% horse serum (HS) and PenStrep for 3 d) at more than 80% confluence. To induce atrophy, the fully differentiated myotubes were treated with 10 μM Dex with or without different concentrations of Ni for 24 h. To check the effect of Ni on myogenesis, compound was treated to C2C12 myoblasts at more than 80% confluence for 48 h. A cancer cachexia model was established by treating 100 ng/ml TNF-α (Sino-biological, China) to fully differentiated myotubes for 24 h, as previously described [19].

### Cell viability assay

Myoblast viability was assessed using the crystal violet assay, which was carried out using the Cold Spring Harbour Protocol of Feoktistova, *et al* [20]. For analysis of myotube viability, the myoblasts were induced to undergo differentiation for 96 h, after which the media was replenished with compound of interest, and the cultures were incubated for a further 48 h. The IC_50_ was calculated based on three runs of the assay.

### Hematoxylin & Eosin (H&E) staining

After removing the culture media, cells were washed with PBS 2 times. 3.7% formaldehyde solution was treated to fix the samples for 15 min at room temperature (RT). After washing cells with PBS for 3 times, 0.2% Triton X-100 solution was used for permeabilization. Cells were then washed with PBS for 3 times and washed with distilled water 2 times. Hematoxylin was treated for 1 min at RT. Cells were washed with tap water to remove hematoxylin and eosin was treated for 45 s. Immersion with 95% and 100% ethanol was used to remove the eosin dye.

### Immunocytochemistry

The Mitotracker dye has been used as a convenient method to stain myotubes in differentiating myoblast cultures [21]. However, Ni has been shown to disrupt mitochondrial membrane potential [13,22]. Therefore, myosin heavy chain immunocytochemistry was used to visualize the myotubes. In accordance with the previously published protocol [9]. After removing culture media, the cells were washed with PBS three times. 3.7% formaldehyde solution was treated to fix the samples by incubation for 15 min at room temperature (RT). After three washes with PBS, 0.2% Triton X-100 solution was used for permeabilization. Cells were washed with PBS for three times and blocked with 1% bovine serum albumin in PBST (0.1% Tween 20 in PBS) for 30 min at RT. Cells were then incubated overnight at 4°C with the primary antibody. Afterwards, the cells were washed with PBS three times and the secondary antibody was treated for 1 h in the dark at RT. Cells were then washed 3 times with PBS in the dark, and counterstained with 1 μM of DAPI in PBS for 1 min at RT. Fluorescence images were captured in 5 different areas using a DMI 3000 B microscope (Leica, Germany) and analysed using ImageJ 1.52 software (National institutes of Health, Bethesda, MD, USA). Myosin heavy chain positive cells containing more than 4 nuclei were defined as myotubes.

### Western blotting

**Fig 1:**
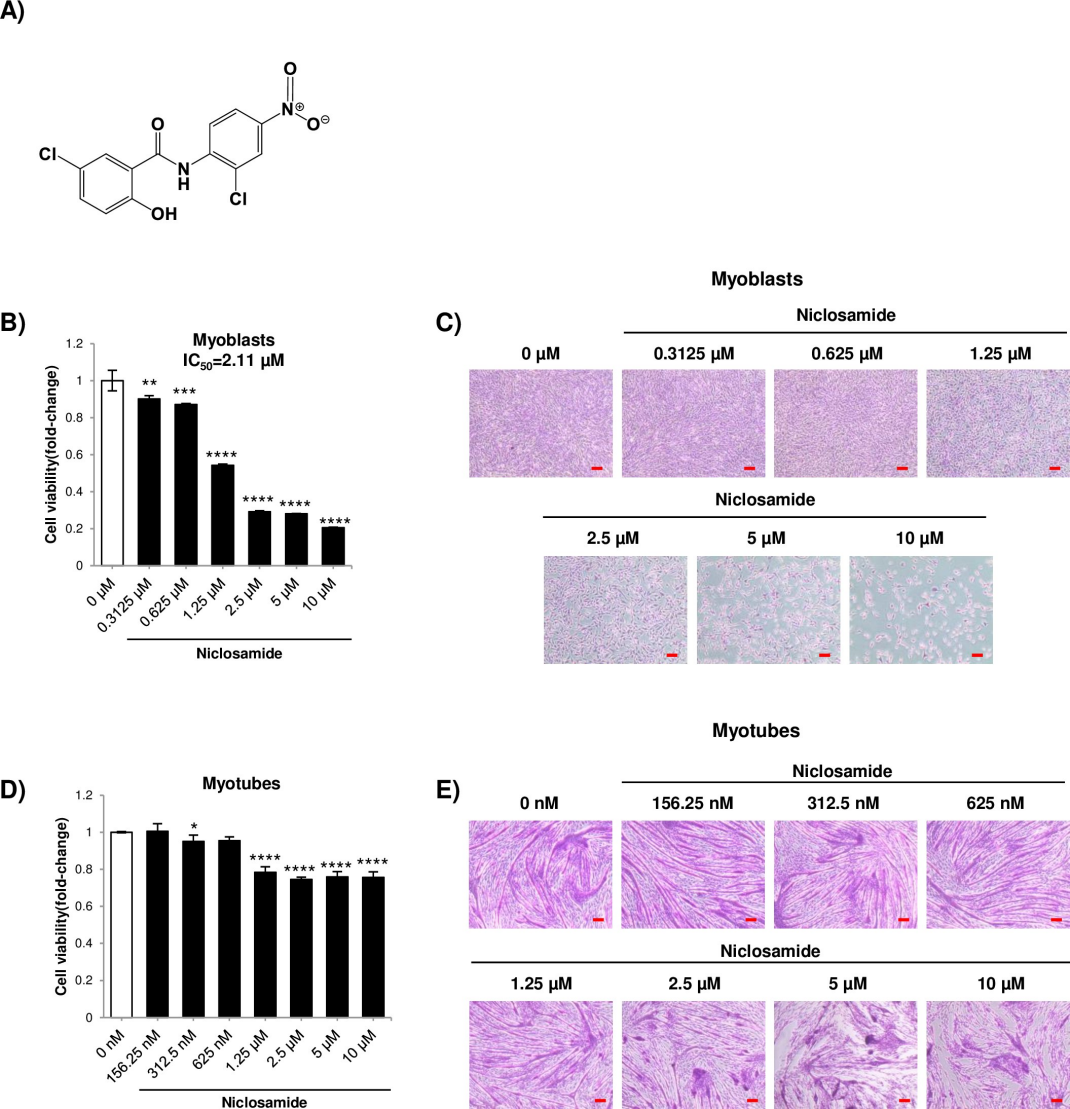
A) Chemical structure of Ni. B) Representative crystal violet assay for C2C12 myoblasts treated with Ni for 48 h (IC_50_ = 2.11 μM). C) Representative images of the treated myoblasts. D) Representative crystal violet assay for C2C12 myotubes treated with Ni. Myoblasts were differentiated for 96 h and treated with Ni for 48 h. E) Representative images of the treated myotubes (scale bar = 100μm). * = *p*<0.05, ** = *p*<0.01, *** = *p*<0.001 and **** = *p*<0.0001 for significantly increased or decreased viability compared to untreated myoblasts or myotubes.

Cell lysate protein concentration was quantified using the Bradford reagent (Bio-Rad, USA, CA). After electrophoresis with 10% SDS-PAGE, separated proteins were transferred onto PVDF membranes (Merck, Germany), blocked with 5% non-fat powdered milk in TBST (0.02% Tween 20 in TBS) and 5% bovine serum albumin in TBST (0.02% Tween 20 in TBS), followed by overnight incubated at 4°C with the primary antibody of interest. The secondary antibody was used at a 1: 10000 dilution, with incubation for 35 min at RT. Densitometry analysis of the gel bands was carried out using ImageJ 1.48 software (National Institutes of Health, Bethesda). Details of the primary and secondary antibodies used in this study are provided in Tables 1 and 2, respectively. The original blot images are shown in Fig 1.

**Table 1:** Primary antibodies used in this study.

Primary antibody	Clone	Company	Catalog No.	Dilution
α-Tubulin	Polyclonal	INVITROGEN	PA5-29444	1:1000
Atrogin-1/MAFbx (F-9)	Monoclonal	SANTA CRUZ	Sc-166806	1:100
MuRF-1	Polyclonal	INVITROGEN	PA5-76695	1:2000
FoxO3a (D19A7)	Monoclonal	CST	#12829	1:1000
Phospho-FoxO3a (Ser253)	Polyclonal	CST	#9466	1:1000
mTOR (7C10)	Monoclonal	Cell Signaling	#2983	1:1000
Phospho-mTOR (Ser2448)	Monoclonal	Cell Signaling	#2971	1:1000
Myosin heavy chain 2 (A4.74)	Monoclonal	SANTA CRUZ	Sc-53095	1:50

**Table 2:** Secondary antibodies used in this study.

Primary antibody	Conjugated use	Company	Catalog No.	Dilution
Goat anti-Mouse IgG (H+L)	HRP	Abcam	ab6789	1:10000
Goat anti-Rabbit IgG HRP	HRP	CST	#7074S	1:2000
Alexa Fluor™ 488 Goat anti-mouse IgG(H+L)	Alexa Fluor 488	INVITROGEN	A11001	1:2000

### Real-time qPCR

The mRNA level of genes of interest was measured using the StepOnePlus Real Time PCR System (Applied Biosystems, UK). cDNA was made from the total RNA using the AccuPower RT PreMix (Bioneer, Republic of Korea). Real-time PCR (qPCR) was conducted according to the manufacturer’s instructions with the following modifications: PCR was performed in triplicate in 20 μL 2X Power SYBR Green PCR Master Mix (Enzynomics, Republic of Korea) composed of 200 nM (final concentration) of the targeted primers and 1 μL of cDNA. Details of the primers used in this study are shown in Table 3. The denaturation step was conducted by incubation for 10 min at 95°C and the amplification step consisted of 40 cycles of denaturation (15 s at 95°C), annealing (1 m at 60°C) and extension (72°C for 20 s) The fluorescence at the extension step was detected at 72°C after each cycle. After the final cycle, melting-point of the samples was analyzed within the range of 60–95°C with fluorescence detection. A specific cDNA sample was included in each run and compared as a reference between runs. The expression level of β-actin was used for normalization while measuring the expression levels of all of the other genes.

**Table 3:** Primers used for qPCR.

Primer name	Primer sequence	Size (bp)	Accession Number
β-Actin	F: TCCCTGGAGAAGAGCTATGAGC R: GAGGTCTTTACGGATGTCAACG	177	NM_007393
Atrogin-1	F: CAGAGAGCTGCTCCGTCTCA R: ACGTATCCCCCGCAGTTTC	178	NM_026346
Murf-1	F: CCGAGTGCAGACGATCATCTC R: TGGAGGATCAGAGCCTCGAT	198	NM_001039048
FoxO3a	F: TGGAGTCCATCATCCGTAGTGA R: CTGGTACCCAGCTTTGAGATGAG	147	NM_019740
Pax7	F: CACAGAGGCAGAGCTGATTGC R: CCAATTGAGGAGAGTGACAGGTT	157	NM_011039
Myf5	F: AGCTGGGCAGAATACGTGCTT R: AGAACAGGCAGAGGAGAATCCA	112	NM_008656
Myod1	F: TGTCCTTTCGAAGCCGTTCT R: TGCAGCCAGAGTGCAAGTG	169	NM_010866
Myogenin	F: AGCGCAGGCTCAAGAAAGTG R: CCGCCTCTGTAGCGGAGAT	181	NM_031189
Myh2	F: GATCACCACGAACCCATATGATT R: TTCATGTTCCCATAATGCATCAC	183	NM_001039545
iNOS	F: CCCCTTCAATGGCTGGTACA R: GCGCTGGACGTCACAGAA	64	NM_010927

### Myotube fusion index

To assess the myogenic effects of the compound, the number of nuclei and Green fluorescent intensity were counted and measured using ImageJ 1.48 software. Myotubes were defined as cells containing more than 4 nuclei.

### Animal model of Dex-induced skeletal muscle atrophy

Animal studies were undertaken out in accordance with the ethical guidelines established by the Animal Care and Use Committee (ACUC) of the Gwangju Institute of Science and Technology, Republic of Korea. The experimental protocol were approved by the ACUC of the Gwangju Institute of Science and Technology, Republic of Korea. In addition to the guidelines established by the ACUC, the study was carried out in compliance with the ARRIVE guidelines. Animals were purchased from Damool Science, Republic of Korea.

Treatment with the glucocorticoid, Dex, has been commonly used to investigate skeletal muscle wasting in animal models [23,24]. 10 week old male C57BL/6J mice were treated with Dex and Ni ethanolamine as follows: 1) Intraperitoneal (IP) delivery of vehicle (DMSO) alone, 2) IP delivery of 25 mg/kg Dex dissolved in vehicle, 3) IP delivery of 25 mg/kg Dex and 20 mg/kg Ni (ethanolamine salt) (n = 5 per group). Mice were treated 7 d and then assessed for muscle condition.

### Grip strength test

Grip strength was measured using a BIO-GS3 (Bioseb, FL, USA). The mice were placed onto the grid with all four paws attached and gently pulled backwards to measure grip until the grid was released. The maximum grip value used to represent muscle force was obtained using 3 trials with an interval of 30 sec.

### Muscle sampling and histological analysis

Mice were anesthetized using ketamine (22 mg/kg; Yuhan, Republic of Korea) and xylazine (10 mg/kg; Bayer, Republic of Korea) in saline by intraperitoneal injection before sacrifice. Quadriceps and gastrocnemius muscles were dissected and weighed. For histological analysis, gastrocnemius muscles were fixed by overnight incubation with 4% paraformaldehyde at 4°C and embedded into Cryo OCT solution kept in -80°C. 8 μm muscle sections were obtained and used for H&E staining with a kit, following the manufacturer’s instructions (Merck, Germany). Cross sectional area was measured with the ImageJ 1.48 software (National Institutes of Health).

### Statistical analysis

Statistical significance was measured using one-way ANOVA (Data Analysis ToolPak for Microsoft Excel 2013). A *p* value of less than 0.05 was considered to be significant. Unless otherwise stated, all data shown are representative of 3 experimental repeats and the graph error bars are standard deviation.

## Results

### Ni treatment in the micromolar concentration range produces cytotoxicity in myoblasts and disrupts myotube morphology

This chemical structure of Ni is shown in Fig 1A. Repurposing studies of Ni are often used at micromolar range [11–13]. The effect of Ni on cell viability in C2C12 myoblasts and myotubes was assessed using the crystal violet assay [20]. At concentrations above 1.25 μM, the viability of C2C12 myoblasts markedly decreased (Fig 1B and 1C). The IC_50_ for cell viability was 2.11 μM. In differentiated myotubes, there was also a marked decrease in myotube viability at 1.25 μM Ni treatment (Fig 1D and 1E).

### Ni treatment does not prevent glucocorticoid-induced skeletal myotube atrophy

**Fig 2:**
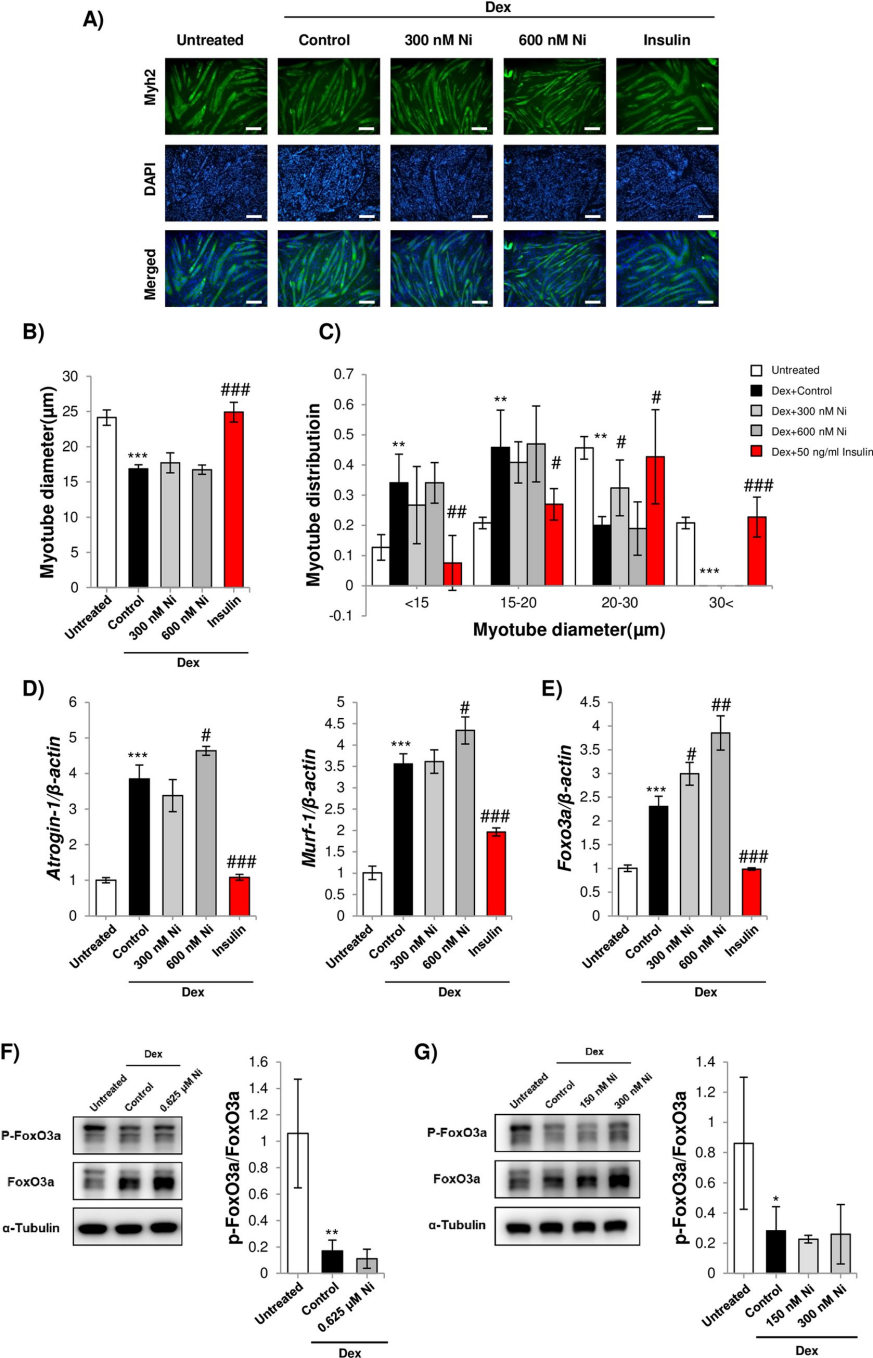
A) Representative images of myosin heavy chain immunostained C2C12 myotubes after treatment with Dex and Ni (scale bar = 100 μm). Myoblasts were differentiated for 72 h and treated with 10 μM Dex, or 10 μM Dex plus 300 nM or 600 nM Ni or 50 ng/ml bovine insulin for 24 h. B) Average diameter of C2C12 myotubes treated with Dex or Dex+Ni. *** = *p*<0.001 indicate significantly decreased compared to the untreated control. ### = *p*<0.001 to indicate significantly increased myotube diameter compared to Dex treated myotubes. C) Myotube diameter distribution. ** = *p*<0.01 and *** = *p*<0.001 indicate significantly decreased or increased proportion of mytube compared to the untreated control. # = *p*<0.05, ## = *p*<0.01 and ### = *p*<0.001 to indicate significantly increased or decreased compared to Dex treated myotubes. D) qPCR analysis of atrogin-1 and MuRF-1 in myoblasts differentiated for 72 h and treated with 10 μM Dex, or 10 μM Dex plus 300 nM, 600 nM Ni or 50 ng/ml bovine insulin for 24 h. E) qPCR analysis of FoxO3a. For A-D) 50 ng/mL insulin treatment was used as a positive control for suppression of Dex-induced myotube atrophy. F-G) Western blot analysis of phosphorylated FoxO3a in myoblasts were differentiated for 72 h and treated with 10 μM Dex, or 10 μM Dex plus 625 nM, 150 nM or 300 nM Ni for 24 h (n = 5 and n = 4). For D-G) * = *p*<0.05, ** = *p*<0.01and *** = *p*<0.001 indicates significantly increased or decreased expression compared to the untreated group. # = *p*<0.05, ## = *p*<0.01, and ### = *p*<0.0001 indicate significantly increased or decreased expression compared to Dex treated myotubes.

The glucocorticoid, Dex, is used to model skeletal muscle atrophy in myotubes [25]. Based on the cell viability data in Fig 1, the effect of Ni on myotube atrophy was assessed at 600 and 300 nM treatment concentrations. Myotube diameter measurements in H&E stained cultures showed no significant effect on average myotube diameter or the proportion of larger myotubes (diameter above 30 μm) (Fig 2A–2C). Expression of the atrogenes, atrogin-1 and MuRF-1 [26], was assessed in myotubes treated with Dex and Ni. 50 ng/mL insulin treatment was used as a positive control for increasing protein synthesis and suppressing atrogene expression [27]. Atrogin-1 and MuRF-1 expression in Dex-treated myotubes was induced by Ni treatment (Fig 2D). Transcription factor Forkhead box O3 (FoxO3a) is a master regulator of the ubiquitin-proteasome pathway of skeletal muscle catabolism [28]. FoxO3a de-phosphorylation induces nuclear translocation from the cytoplasm and upregulation of target atrogenes. Ni treatment did not significantly reduce FoxO3a phosphorylation or expression in Dex-treated myotubes (Fig 2E–2G).

### Ni treatment alone induces myotube atrophy

**Fig 3:**
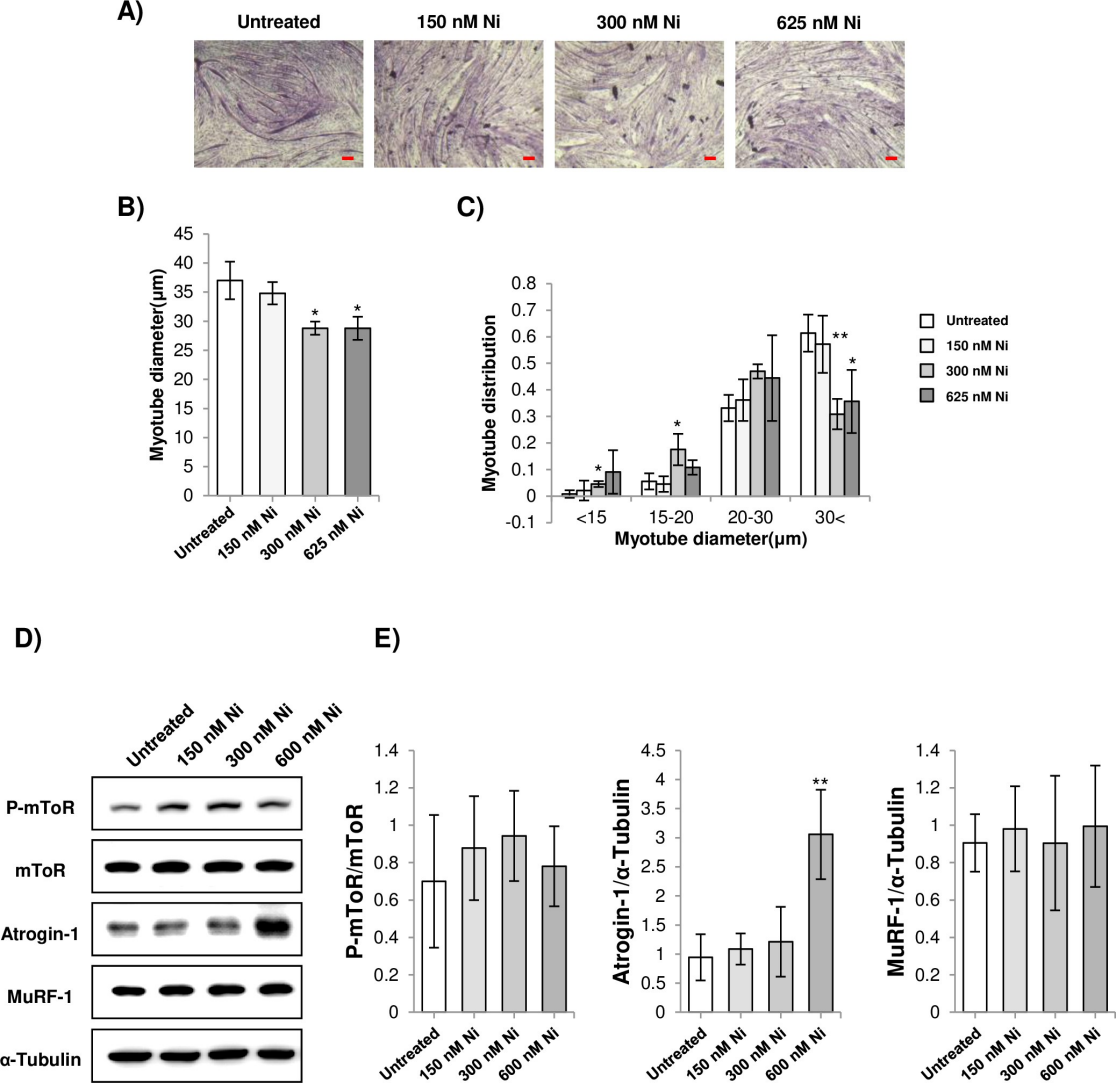
A) Representative images of H&E stained C2C12 myotubes treated with Ni (scale bar = 100 μm). Myoblasts were differentiated for 72 h and treated with 150 nM, 300 nM, and 625 nM Ni for 24 h. B-C) Average diameter and myotube diameter distribution of C2C12 myotubes treated with Ni. * = *p*<0.05 and ** = *p*<0.01 to indicate significantly decreased or increased compared to the untreated control. D) Western blot analysis of atrogin-1, MuRF-1, mTOR and phosphorylated mTOR expression. E) Densitometry analysis of atrogin-1, MuRF-1, mTOR and phosphorylated mTOR expression. ** = *p*<0.01 indicates significantly decreased or increased expression compared to the untreated control (n = 4).

In consequence of the finding that Ni treatment does not inhibit experimentally induced myotube atrophy, the effect of Ni treatment alone on myotube atrophy was investigated. Ni treatment produced myotube atrophy in a concentration-dependent manner, as indicated by decreased average myotube diameter and the proportion of myotubes with relatively larger diameters (above 30 μm) (Fig 3A–3C). Mammalian target of rapamycin (mTOR) is a key regulator of skeletal muscle mass [29]. mTOR phosphorylation in Ni-treated myotubes was assessed by western blotting. It was observed that mTOR phosphorylation was not significantly affected by Ni treatment (Fig 3D and 3E). In addition, increasing concentrations of Ni treatment induced the expression of atrogin-1 in the myotubes, although there was no effect on MuRF-1 expression (Fig 3D and 3E).

### Ni treatment inhibits myoblast differentiation

**Fig 4:**
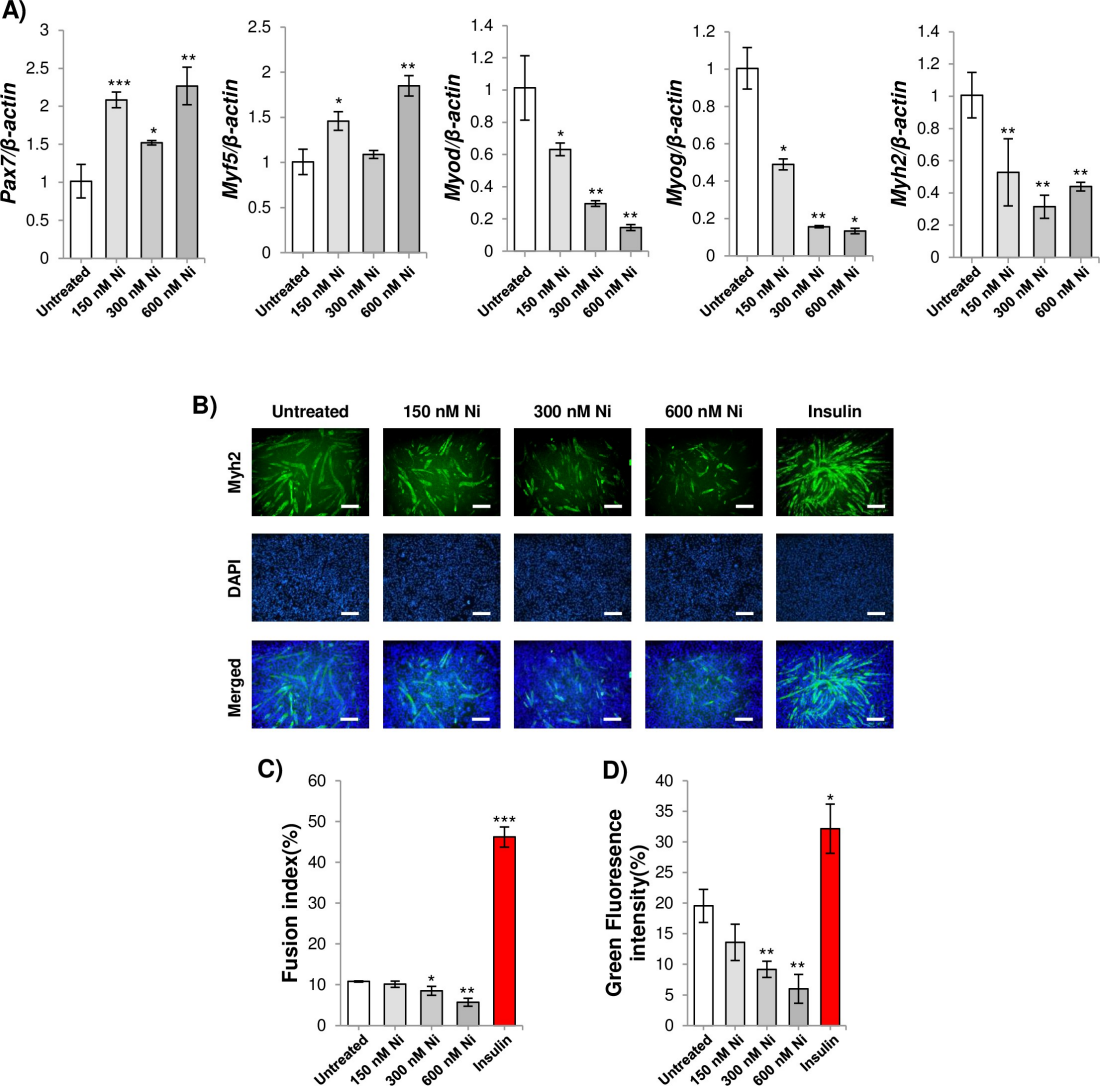
A) qPCR analysis of *Pax7*, *Myf5*, *Myod*, *Myog* and *Myh2* expression in differentiating C2C12 myoblasts treated with 150 nM, 300 nM or 600 nM Ni for 24 h. * = *p*<0.05, ** = *p*<0.01 and *** = *p*<0.001 indicate significantly increased or decreased expression compared to untreated myoblasts. B) Representative images of myosin heavy chain immunostained C2C12 cultures after treatment with 150 nM, 300 nM,600 nMNi and 50 ng/ml bovine insulin for 48 h (the size bar = 100 μm). Myotubes are shown using white arrows. C) Fusion index (DAPI counts in myosin heavy chain 2 stained myotubes/total DAPI counts). 50 ng/mL insulin treatment was used as a positive control for enhancement of myogenesis. * = *p*<0.05, ** = *p*<0.01 and *** = *p*<0.001 for significant difference compared to untreated myoblasts. D) Green fluorescent intensity was measured by using image J. * = *p*<0.05 and ** = *p*<0.01 for significant difference compared to untreated myoblasts.

The capacity of skeletal muscle to regenerate decreases with old age [30]. To assess the effects of Ni on regeneration, C2C12 myoblasts were treated with compound and differentiated into myotubes. The genetic program regulating differentiation involves downregulation of the myoblast markers *Myf5* and *Pax7*, and upregulation of the master myogenesis factors *Myog* and *Myod* [31]. Nanomolar Ni treatment increased the expression of *Myf5* and *Pax7* in myoblasts cultured in differentiation media (Fig 4A). In addition, expression of *Myog*, *Myod* and the myotube structural protein, *Myh2*, expression was inhibited by Ni. The negative effect of Ni on myogenesis was confirmed by measuring myosin heavy chain immunostained myotubes (Fig 4B–4D).

### Ni does not prevent skeletal muscle atrophy in an animal model of Dex-induced muscle wasting

**Fig 5:**
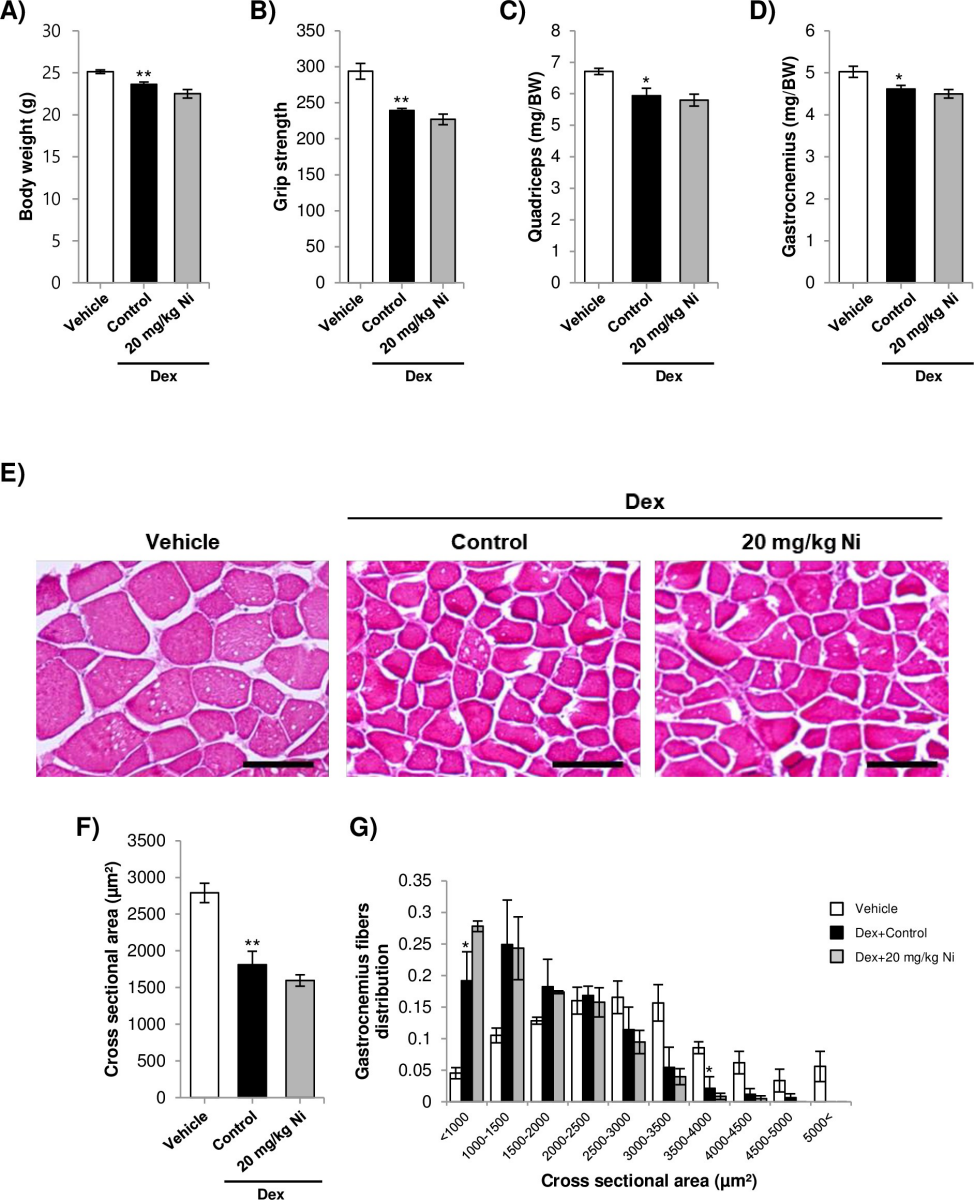
A) Body weight in mice treated with dexamethasone, dexamethasone and niclosamide, or vehicle alone. B) Grip strength in the treated mice. C-D) Quadriceps and gastrocnemius muscle mass in the treated mice. For A-D) * = *p*<0.05 and ** = *p*<0.01, indicate significantly decreased compared to the vehicle control. E) Representative images of H&E stained gastrocnemius muscle. Scale bar = 100 μm F) Average cross sectional area in the gastrocnemius muscle. ** = *p*<0.01 for decreased average cross sectional area compared to the vehicle mice. G) Fiber cross sectional area distribution in the gastrocnemius muscle. * = *p*<0.05 for decreased or increased proportion of fibers compared to untreated mice.

The effect of Ni on skeletal muscle atrophy *in vivo* was assessed using the Dex model, as previously described [32,33]. Body weight was decreased by Dex treatment and not significantly affected by Ni (Fig 5A). Dex treatment also reduced grip strength, and was not significantly affected by Ni (Fig 5B). Quandriceps and gastrocnemius mass was decreased by Dex treatment and not significantly affected by Ni (Fig 5C and 5D). Histological analysis of the gastrocnemius muscle indicated that Dex treatment reduced both the muscle fiber cross sectional area and the proportion of larger-sized fibers, and these parameters were not significantly recovered by Ni treatment (Fig 5E–5G).

### Ni is more cytotoxic for myoblasts than cancer cells and is not effective in a model of cancer cachexia

**Fig 6:**
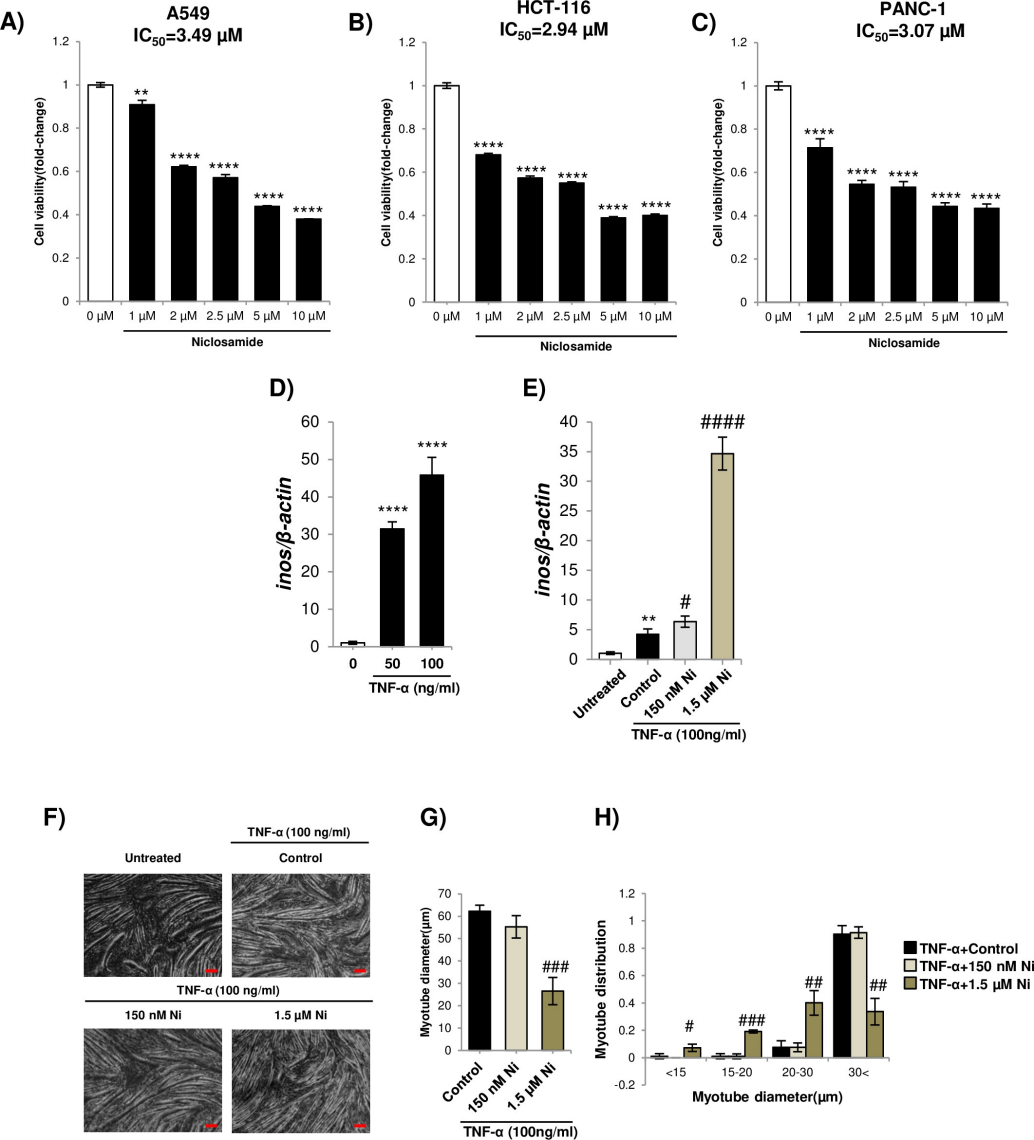
A-C) Representative crystal violet assay for A549, HCT-116, and PANC-1 cells treated with Ni for 48 h. The IC_50_ 3.49 μM, 2.94 μM, and 3.07 μM, respectively. ** = *p*<0.01 and **** = *p*<0.0001 indicate significantly decreased viability compared to the untreated control. D) qPCR analysis of iNOS expression in C2C12 myotubes treated with 50 or 100 ng/mL TNF-α for 24 h. **** = *p*<0.0001 indicate significantly decreased viability compared to the untreated control. E) qPCR analysis of iNOS expression in C2C12 myotubes treated with 100 ng/mL TNF-α and 150 nM, or 1.5 μM, Ni for 24 h. ** = *p*<0.01 indicates significantly increased expression of iNOS compared to the untreated control. # = *p*<0.05 and #### = *p*<0.0001 indicate significantly increased expression of iNOS compared to the 100 ng/mL TNF-alpha control. F) Representative DIC images of untreated C2C12 myotubes or myotubes treated with 100 ng/mL TNF-α and 150 nM, or 1.5 μM, Ni for 24 h. (the size bar = 100 μm). G-H) Average myotube diameter and myotube diameter distribution. # = *p*<0.05, ## = *p*<0.01 and ### = *p*<0.001 indicate significantly increased or decreased compared to the untreated control.

Ni is a known anti-cancer drug. Cancer-induced skeletal muscle atrophy (cancer cachexia) is most commonly associated with pancreatic, colon and lung carcinoma [34]. To assess if Ni can preferentially kill cachexia-associated cancer cells compared to skeletal myoblasts, the IC_50_ for cytotoxicity in A549 (human lung adenocarcinoma), HCT-116 (human colorectal carcinoma), and PANC1 (human pancreatic ductal carcinoma) cells was measured using the crystal violet assay of cell viability. The IC_50_ for cytotoxicity was 3.49 μM, 2.94 μM and 3.07 μM, respectively (Fig 6A–6C).

Myotubes treated with TNF-α (formerly termed cachectin) have also been used to model cancer cachexia [19]. Inducible nitric oxide synthase (iNOS) in skeletal muscle is a key mediator cancer cachexia [35]. Treatment with a high or low concentration of Ni (150 nM or 1.5 μM) significantly increase *iNOS* expression in myotubes exposed to TNF-α (Fig 6D and 6E). It was observed that 1.5 μM Ni treatment reduced average myotube diameter and the proportion of wider myotubes (Fig 6F–6H).

## Discussion

In this study, we investigated whether Ni, a drug used in repurposing applications for numerous diseases, has therapeutic potential to treat muscle atrophy. Cell-based assays indicated that Ni treatment could not prevent myotube atrophy and inhibited myogenesis. The IC_50_ for Ni in myoblasts was found to be lower than for cancer cells and treatment did not prevent the induction of *iNOS*, a major mediator of muscle atrophy, in a myotube-based model of cancer cachexia. To our knowledge, this is the first investigation of Ni as a possible drug candidate for glucocorticoid-induced muscle or cancer cachexia.

Ni is known to have poor oral bioavailability and is primarily detected in the liver and kidney [13,36]. It was reported that at peak plasma concentration, Ni could not be detected in skeletal muscle [13]. Our rationale for testing Ni *in vitro* was based on the possibility of repurposing Ni as a drug for sarcopenia that could be delivered by injection, rather than oral delivery. Due to the lack of effective drugs for sarcopenia, some clinical trials have tested injectable compounds, such as landogrozumab or follistatin, that can be delivered subcutaneously or intravenously [37]. However, we also believe that it would have been preferable to develop Ni as an orally bioavailable drug for muscle wasting disorders. Therefore, if we had discovered Ni to be effective at preventing myotube atrophy *in vitro*, we could have tested orally bioavailable derivatives of Ni in a follow-up study, which has been previously described for anti-cancer studies of Ni [38]. In addition, *in vivo* studies have utilized microencapsulated formulations of Ni, such as loaded submicron lipid emulsions, which have shown improved oral bioavailability [39].

Concerning the mechanism of cellular uptake for niclosamide, based on the molecular weight of niclosamide (MW 327.12) it is classified as a small molecule compound, which allows the possibility of rapid diffusion across cell membranes to reach intracellular sites of action [40,41]. In addition, niclosamide has been reported to be a cell-permeable salicylanilide drug [40]. For previous studies in which niclosamide was encapsulated in liposomes to increase bioavailability, the mechanism of cell entry is most likely via endocytotic processes, although cationic liposomes that can undergo lipid mixing with cellular membranes [42,43]. A previous study has also indicated that niclosamide can reach concentrations of 0.25–6.0 μg/mL in the serum of humans after a 2,000 mg single dose [44]. Furthermore, a niclosamide concentration of 0.1 μg/g in the skeletal muscle of livestock has been reported 28 h after a single dose of 80 mg/kg body weight [44].

Our analysis also showed that Ni treatment alone induced myotube atrophy, did not increase mTOR phosphorylation and increased the expression of atrogin-1. This inability of Ni to increase mTOR phosphorylation may be due to mitochondrial uncoupling, which has been showed to increase with aging in skeletal muscle [45]. Ni has also been shown to modify cytoplasmic pH to inhibit mTOR signalling and the negative effect of Ni on Wnt signalling may perturb skeletal muscle maintenance [46,47]. Further studies may focus on identifying which cellular mechanism is directly responsible for the effects of Ni on regulators of myotube atrophy. Our results indicate that Ni can inhibit myoblast differentiation. Ni is known to disrupt the Wnt/β-catenin pathway, which regulates myoblast fusion [14,48,49]. In addition, Ni suppresses STAT3 signalling in myoblasts, which has been shown to enhance Pax7 expression, promote premature differentiation, and improve regeneration after skeletal muscle injury *in vivo* [14,50,51]. Therefore, we speculate that the inhibition of Wnt/β-catenin signalling is responsible for the anti-myogenesis effect. The pathological activation of STAT3 signalling also promotes myotube and skeletal muscle atrophy [51–53]. In contrast, the activation of Frizzled receptors via Wnt/β-catenin stimulates PI3K (phosphatidylinositol 3-kinase) signalling and the AKT/mTOR pathway, which increases protein synthesis. Thus, we hypothesize that inhibition of Wnt/β-catenin signalling by Ni may also produce the myotube atrophy observed in this study. We used the glucocorticoid, dexamethasone, to produce a model of skeletal muscle atrophy, as previously described [23,24]. Dexamethasone induces muscle atrophy by suppression of the insulin-like growth factor 1 (IGF-1)/Akt/PI3K signalling pathway [54,55]. However, previous research has shown that niclosamide treatment does not activate the IGF-1/Akt/PI3K signalling pathway [56,57]. Niclosamide treatment inhibited this pathway in these studies. Therefore, even though niclosamide can suppress STAT3 signalling, this will not prevent muscle atrophy induced by agents such as dexamethasone.

α-Tubulin was used as an internal control for Western blotting and densitometry analysis (Figs 2F, 2G and 3D). However, it should be noted that it is unsuitable as a loading and transfer control, because of the high expression levels of housekeeping genes, such as α-tubulin, β-tubulin and GAPDH. Large amounts of these protein are loaded to detect low-abundance target proteins, which would prevent an accurate quantification of the housekeeping gene expression. Thus, caution should be employed when interpreting protein expression relative to α-tubulin. The quality of the antibodies for the housekeeping genes is also an important factor in Western blotting.

Ni treatment has been previously assessed in a model of doxorubicin-induced skeletal muscle atrophy. Zhan *et al*, reported that oral delivery of Ni ethanolamine improves functional parameters, such as grip strength, and inhibits the induction of FoxO3a atrogene expression [58]. These results are in contrast with the data presented in the current study. We speculate that this discrepancy may be due to the different mechanisms by which Dex and doxorubicin produce muscle atrophy. Doxorubicin has been reported to increase p38 MAPK-FoxO3a signalling to induce atrophy [59]. Muscle atrophy produced by Dex has been linked to the suppression of insulin-like growth factor (IGF)—phosphoinositide 3-kinase—Akt signalling [60]. We hypothesize that Ni treatment can suppress p38-MAPK signalling to prevent muscle atrophy produced by doxorubicin, but cannot reverse the effects of Dex on IGF signalling and muscle atrophy.

Cancer cachexia is the severe muscle atrophy that occurs in some cancer patients with advanced metastatic disease, especially pancreatic, colorectal and lung carcinoma [34]. Cancer cachexia significantly impacts on patient quality of life, response to chemotherapy and mortality. Ni has been shown to possess anti-cancer activity in animal models [10]. The data presented in our study suggests that Ni may inhibit skeletal muscle regeneration. In addition, the IC_50_ for skeletal myoblast cytotoxicity was lower than in cancer cells. Previous studies of Ni anti-cancer activity have tended to utilize cancer cell xenograft models [61], including patient derived xenografts [62]. These models may not fully recapitulate cancer cachexia, which is observed in advanced metastasis and requires a fully functional immune system [34]. It may be interesting to test Ni anti-cancer activity and skeletal muscle integrity in the primary tumor resection model, which produces cancer cachexia with greater similarity to that observed in patients with advanced metastasis [34].

Although our results suggest that Ni is unsuitable for repurposing to treat Dex-induced muscle atrophy or cancer cachexia, we did observe interesting biological activities. Ni prevented myogenesis by blocking the downregulation of *Pax7* and *Myf5* in myoblasts incubated with low serum differentiation media. These genes maintain the undifferentiated state in satellite cells that reside next to differentiated muscle fibres *in vivo* [31]. Consequently, induction of the ‘master’ transcriptional regulators of myogenesis, *Myod* and *Myog*, was suppressed along with the synthesis of muscle fibre structural proteins, as shown by reduced levels of *Myh2* in Ni treated myoblasts. Ni treatment may also enhance the catabolic program in myotubes, as indicated by decreased diameter in myotubes cultured with Dex or TNF-α. We propose that Ni could be utilized as a chemical tool to study cellular mechanisms regulating these pivotal pathways in muscle cells.

## Supporting information

S1 File Original blot images. Raw membrane revision pptx.
(PPTX)

S2 File PLoS one Ni muscle atrophy western quantification. (XLSX)
