# Supplementary material for: Investigation of niclosamide as a repurposing agent for skeletal muscle atrophy
Source: PLoS One. 2021 May 26;16(5):e0252135. doi: 10.1371/journal.pone.0252135 (PMC8153455; doi:10.1371/journal.pone.0252135)
Supplement: S1 File — Raw membrane revision pptx. (PPTX) [file pone.0252135.s001.pptx]

## Slide 1
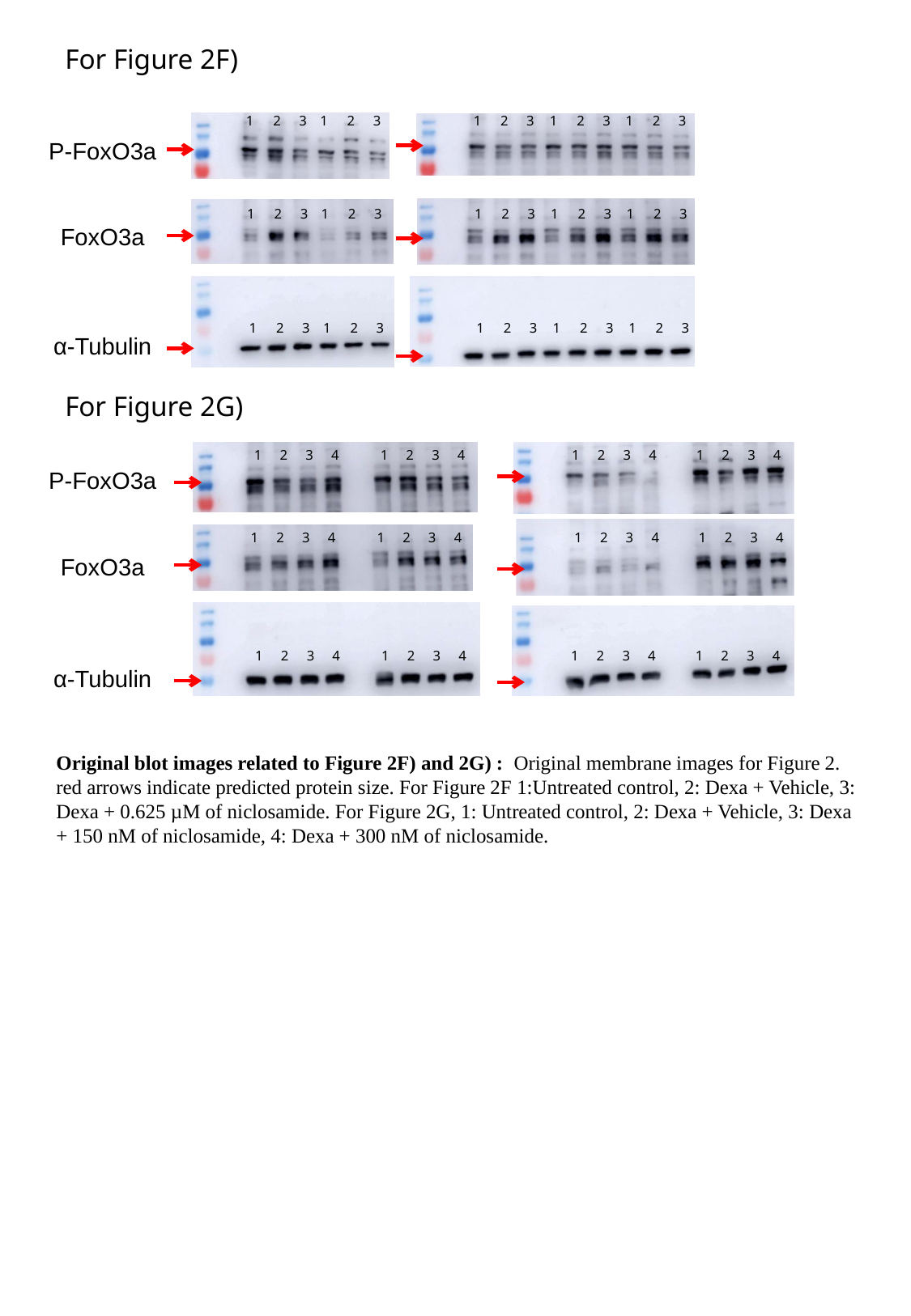

For Figure 2F)
1
2
3
1
2
3
1
2
3
1
2
3
1
2
3
P-FoxO3a
1
2
3
1
2
3
1
2
3
1
2
3
1
2
3
FoxO3a
1
2
3
1
2
3
1
2
3
1
2
3
1
2
3
α-Tubulin
For Figure 2G)
1
2
3
4
1
2
3
4
1
2
3
4
1
2
3
4
P-FoxO3a
1
2
3
4
1
2
3
4
1
2
3
4
1
2
3
4
FoxO3a
1
2
3
4
1
2
3
4
1
2
3
4
1
2
3
4
α-Tubulin
Original blot images related to Figure 2F) and 2G) : Original membrane images for Figure 2. red arrows indicate predicted protein size. For Figure 2F 1:Untreated control, 2: Dexa + Vehicle, 3: Dexa + 0.625 µM of niclosamide. For Figure 2G, 1: Untreated control, 2: Dexa + Vehicle, 3: Dexa + 150 nM of niclosamide, 4: Dexa + 300 nM of niclosamide.

## Slide 2
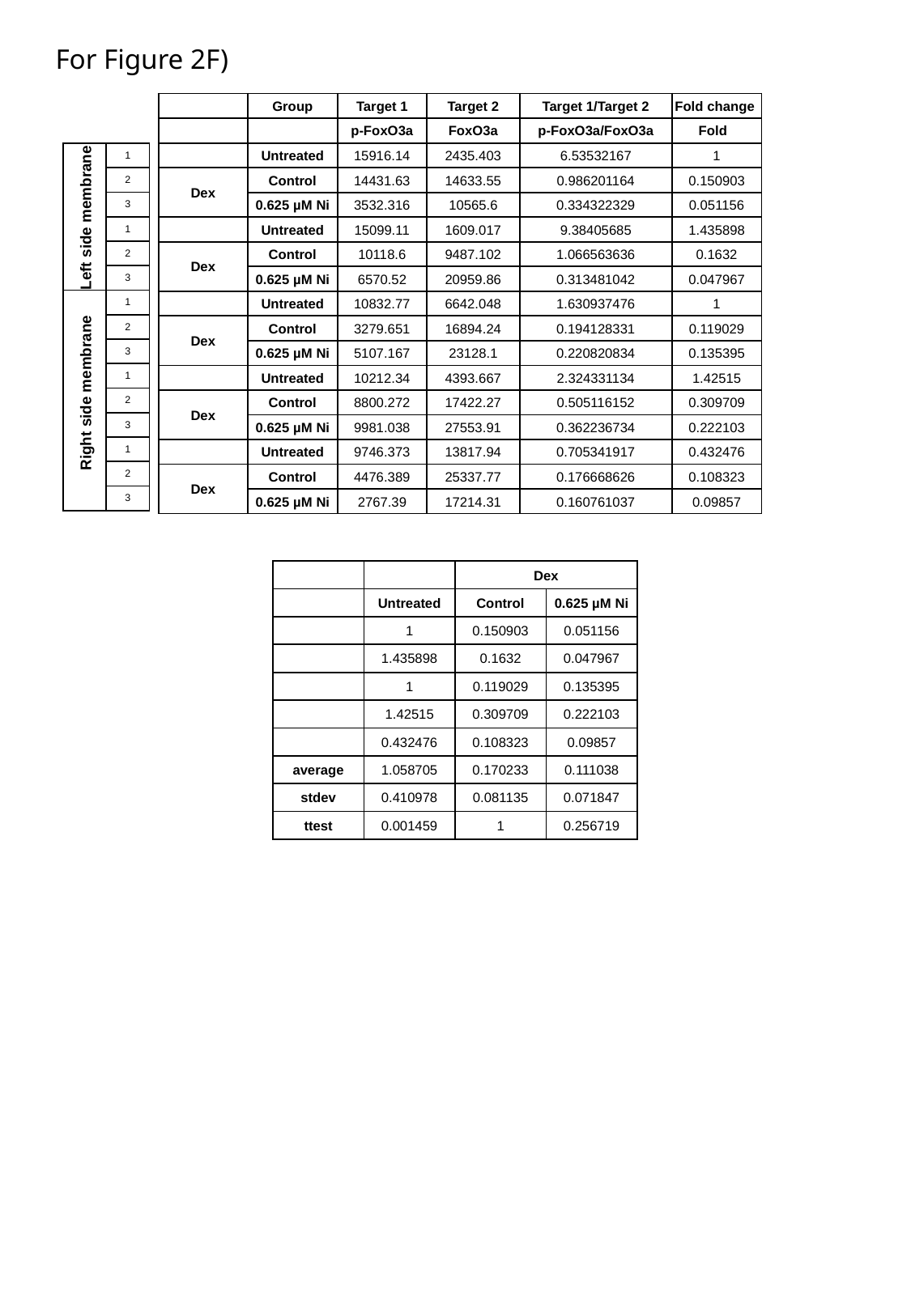

For Figure 2F)
| | Group | Target 1 | Target 2 | Target 1/Target 2 | Fold change |
| --- | --- | --- | --- | --- | --- |
| | | p-FoxO3a | FoxO3a | p-FoxO3a/FoxO3a | Fold |
| | Untreated | 15916.14 | 2435.403 | 6.53532167 | 1 |
| Dex | Control | 14431.63 | 14633.55 | 0.986201164 | 0.150903 |
| | 0.625 µM Ni | 3532.316 | 10565.6 | 0.334322329 | 0.051156 |
| | Untreated | 15099.11 | 1609.017 | 9.38405685 | 1.435898 |
| Dex | Control | 10118.6 | 9487.102 | 1.066563636 | 0.1632 |
| | 0.625 µM Ni | 6570.52 | 20959.86 | 0.313481042 | 0.047967 |
| | Untreated | 10832.77 | 6642.048 | 1.630937476 | 1 |
| Dex | Control | 3279.651 | 16894.24 | 0.194128331 | 0.119029 |
| | 0.625 µM Ni | 5107.167 | 23128.1 | 0.220820834 | 0.135395 |
| | Untreated | 10212.34 | 4393.667 | 2.324331134 | 1.42515 |
| Dex | Control | 8800.272 | 17422.27 | 0.505116152 | 0.309709 |
| | 0.625 µM Ni | 9981.038 | 27553.91 | 0.362236734 | 0.222103 |
| | Untreated | 9746.373 | 13817.94 | 0.705341917 | 0.432476 |
| Dex | Control | 4476.389 | 25337.77 | 0.176668626 | 0.108323 |
| | 0.625 µM Ni | 2767.39 | 17214.31 | 0.160761037 | 0.09857 |
| | 1 |
| --- | --- |
| | 2 |
| | 3 |
| | 1 |
| | 2 |
| | 3 |
| | 1 |
| | 2 |
| | 3 |
| | 1 |
| | 2 |
| | 3 |
| | 1 |
| | 2 |
| | 3 |
Left side membrane
Right side membrane
| | | Dex | |
| --- | --- | --- | --- |
| | Untreated | Control | 0.625 µM Ni |
| | 1 | 0.150903 | 0.051156 |
| | 1.435898 | 0.1632 | 0.047967 |
| | 1 | 0.119029 | 0.135395 |
| | 1.42515 | 0.309709 | 0.222103 |
| | 0.432476 | 0.108323 | 0.09857 |
| average | 1.058705 | 0.170233 | 0.111038 |
| stdev | 0.410978 | 0.081135 | 0.071847 |
| ttest | 0.001459 | 1 | 0.256719 |

## Slide 3
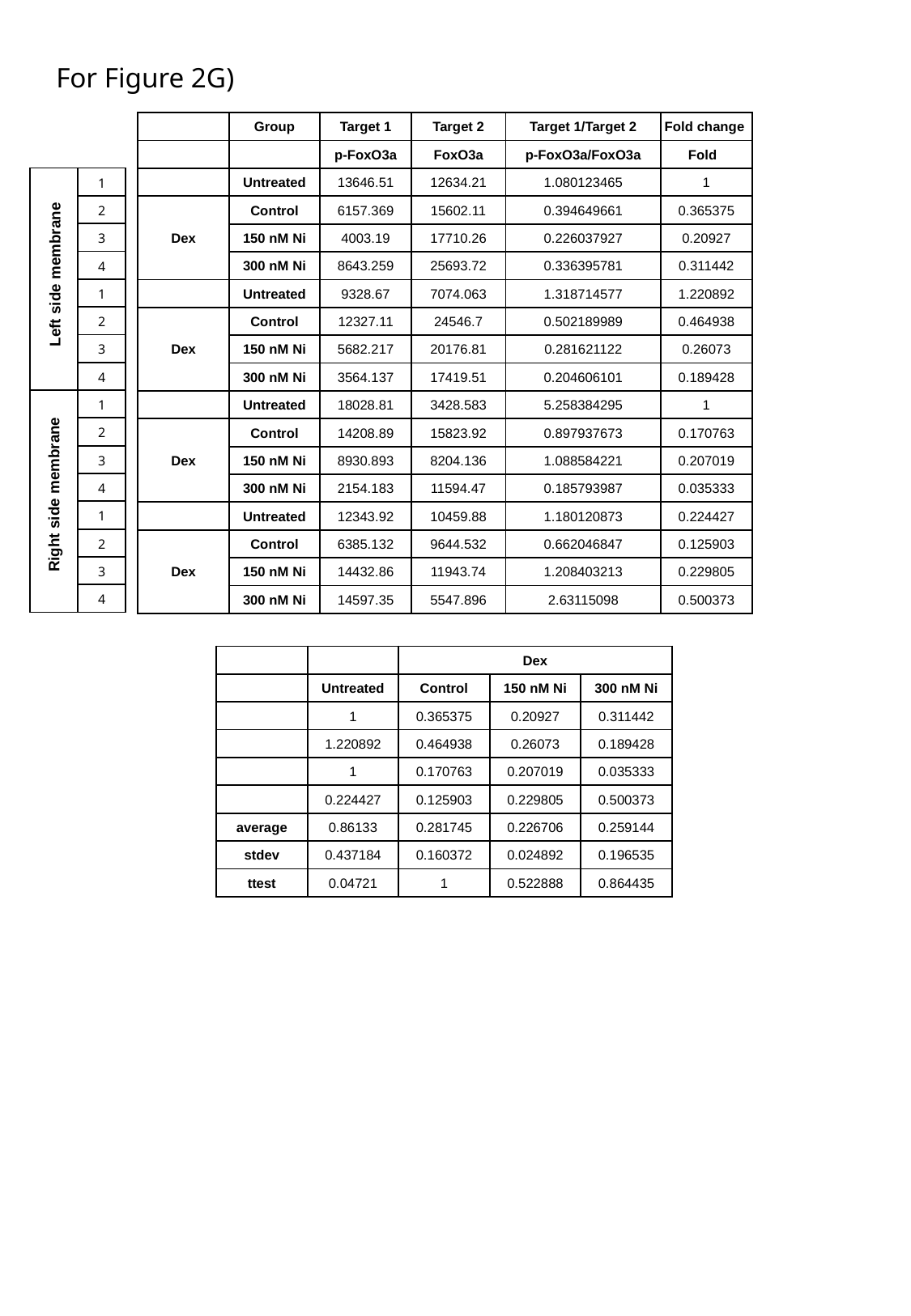

For Figure 2G)
| | Group | Target 1 | Target 2 | Target 1/Target 2 | Fold change |
| --- | --- | --- | --- | --- | --- |
| | | p-FoxO3a | FoxO3a | p-FoxO3a/FoxO3a | Fold |
| | Untreated | 13646.51 | 12634.21 | 1.080123465 | 1 |
| Dex | Control | 6157.369 | 15602.11 | 0.394649661 | 0.365375 |
| | 150 nM Ni | 4003.19 | 17710.26 | 0.226037927 | 0.20927 |
| | 300 nM Ni | 8643.259 | 25693.72 | 0.336395781 | 0.311442 |
| | Untreated | 9328.67 | 7074.063 | 1.318714577 | 1.220892 |
| Dex | Control | 12327.11 | 24546.7 | 0.502189989 | 0.464938 |
| | 150 nM Ni | 5682.217 | 20176.81 | 0.281621122 | 0.26073 |
| | 300 nM Ni | 3564.137 | 17419.51 | 0.204606101 | 0.189428 |
| | Untreated | 18028.81 | 3428.583 | 5.258384295 | 1 |
| Dex | Control | 14208.89 | 15823.92 | 0.897937673 | 0.170763 |
| | 150 nM Ni | 8930.893 | 8204.136 | 1.088584221 | 0.207019 |
| | 300 nM Ni | 2154.183 | 11594.47 | 0.185793987 | 0.035333 |
| | Untreated | 12343.92 | 10459.88 | 1.180120873 | 0.224427 |
| Dex | Control | 6385.132 | 9644.532 | 0.662046847 | 0.125903 |
| | 150 nM Ni | 14432.86 | 11943.74 | 1.208403213 | 0.229805 |
| | 300 nM Ni | 14597.35 | 5547.896 | 2.63115098 | 0.500373 |
| | 1 |
| --- | --- |
| | 2 |
| | 3 |
| | 4 |
| | 1 |
| | 2 |
| | 3 |
| | 4 |
| | 1 |
| | 2 |
| | 3 |
| | 4 |
| | 1 |
| | 2 |
| | 3 |
| | 4 |
Left side membrane
Right side membrane
| | | Dex | | |
| --- | --- | --- | --- | --- |
| | Untreated | Control | 150 nM Ni | 300 nM Ni |
| | 1 | 0.365375 | 0.20927 | 0.311442 |
| | 1.220892 | 0.464938 | 0.26073 | 0.189428 |
| | 1 | 0.170763 | 0.207019 | 0.035333 |
| | 0.224427 | 0.125903 | 0.229805 | 0.500373 |
| average | 0.86133 | 0.281745 | 0.226706 | 0.259144 |
| stdev | 0.437184 | 0.160372 | 0.024892 | 0.196535 |
| ttest | 0.04721 | 1 | 0.522888 | 0.864435 |

## Slide 4
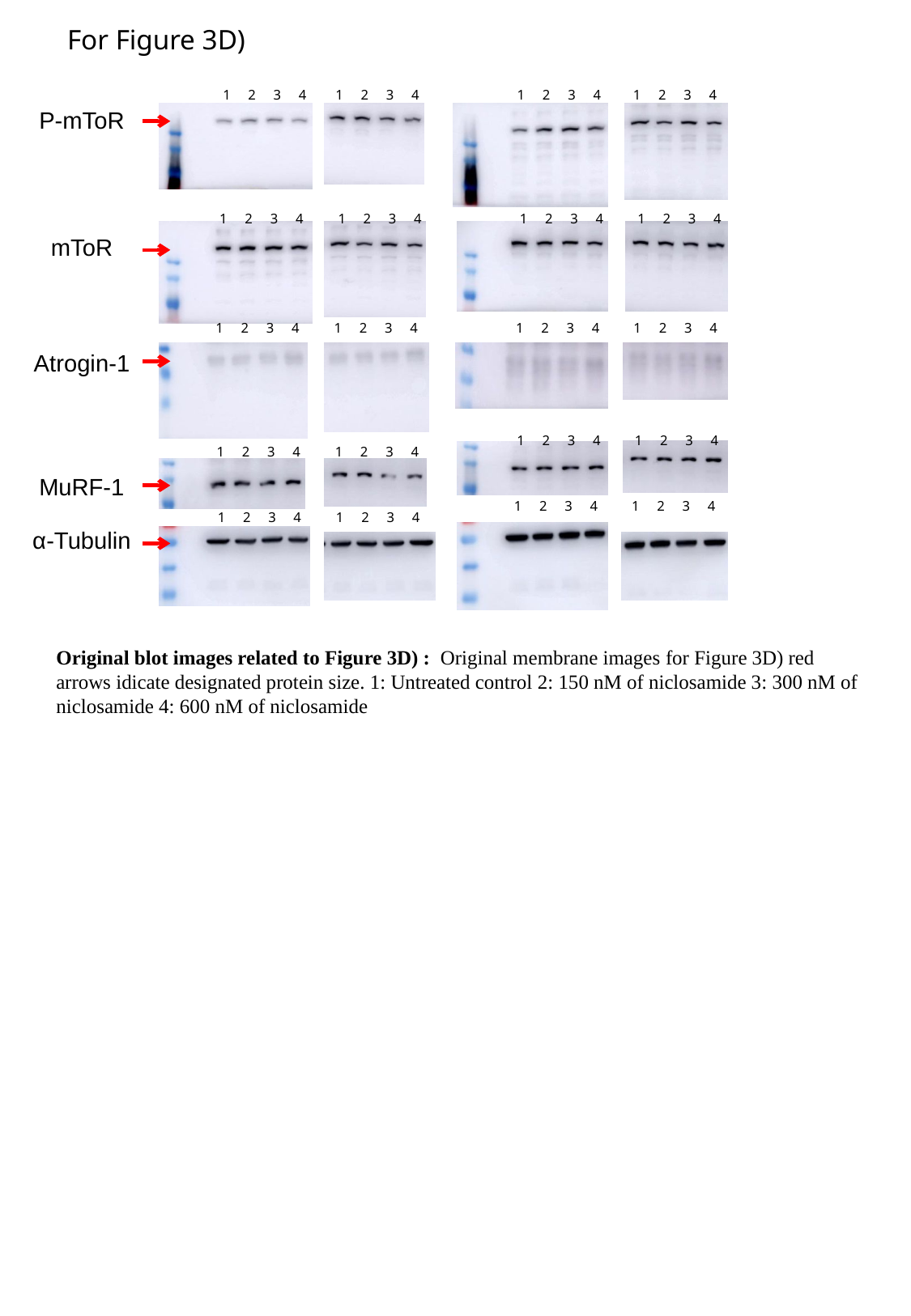

For Figure 3D)
1
2
3
4
1
2
3
4
1
2
3
4
1
2
3
4
P-mToR
1
2
3
4
1
2
3
4
1
2
3
4
1
2
3
4
mToR
1
2
3
4
1
2
3
4
1
2
3
4
1
2
3
4
Atrogin-1
1
2
3
4
1
2
3
4
1
2
3
4
1
2
3
4
MuRF-1
1
2
3
4
1
2
3
4
1
2
3
4
1
2
3
4
α-Tubulin
Original blot images related to Figure 3D) : Original membrane images for Figure 3D) red arrows idicate designated protein size. 1: Untreated control 2: 150 nM of niclosamide 3: 300 nM of niclosamide 4: 600 nM of niclosamide

## Slide 5
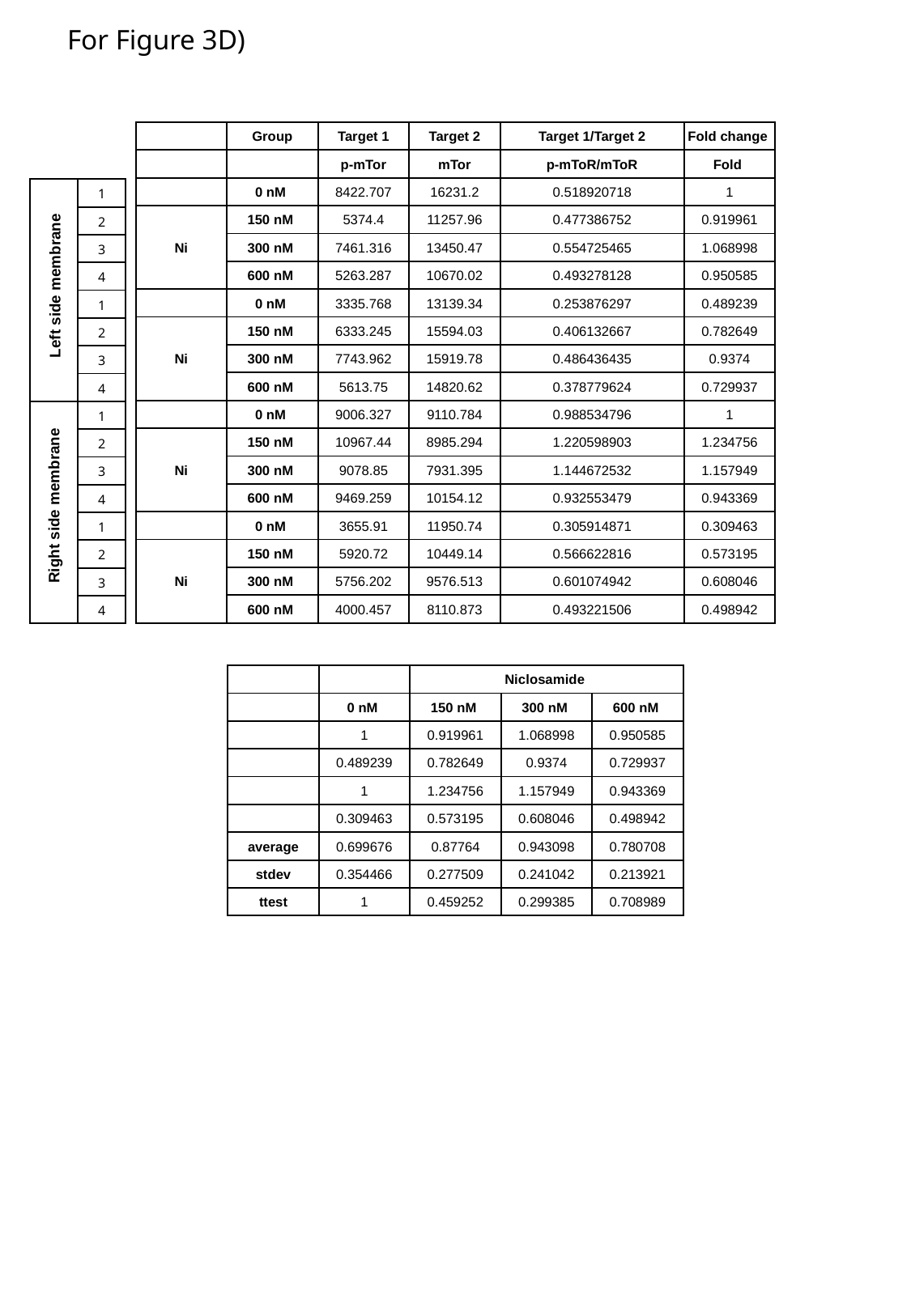

For Figure 3D)
| | Group | Target 1 | Target 2 | Target 1/Target 2 | Fold change |
| --- | --- | --- | --- | --- | --- |
| | | p-mTor | mTor | p-mToR/mToR | Fold |
| | 0 nM | 8422.707 | 16231.2 | 0.518920718 | 1 |
| Ni | 150 nM | 5374.4 | 11257.96 | 0.477386752 | 0.919961 |
| | 300 nM | 7461.316 | 13450.47 | 0.554725465 | 1.068998 |
| | 600 nM | 5263.287 | 10670.02 | 0.493278128 | 0.950585 |
| | 0 nM | 3335.768 | 13139.34 | 0.253876297 | 0.489239 |
| Ni | 150 nM | 6333.245 | 15594.03 | 0.406132667 | 0.782649 |
| | 300 nM | 7743.962 | 15919.78 | 0.486436435 | 0.9374 |
| | 600 nM | 5613.75 | 14820.62 | 0.378779624 | 0.729937 |
| | 0 nM | 9006.327 | 9110.784 | 0.988534796 | 1 |
| Ni | 150 nM | 10967.44 | 8985.294 | 1.220598903 | 1.234756 |
| | 300 nM | 9078.85 | 7931.395 | 1.144672532 | 1.157949 |
| | 600 nM | 9469.259 | 10154.12 | 0.932553479 | 0.943369 |
| | 0 nM | 3655.91 | 11950.74 | 0.305914871 | 0.309463 |
| Ni | 150 nM | 5920.72 | 10449.14 | 0.566622816 | 0.573195 |
| | 300 nM | 5756.202 | 9576.513 | 0.601074942 | 0.608046 |
| | 600 nM | 4000.457 | 8110.873 | 0.493221506 | 0.498942 |
| | 1 |
| --- | --- |
| | 2 |
| | 3 |
| | 4 |
| | 1 |
| | 2 |
| | 3 |
| | 4 |
| | 1 |
| | 2 |
| | 3 |
| | 4 |
| | 1 |
| | 2 |
| | 3 |
| | 4 |
Left side membrane
Right side membrane
| | | Niclosamide | | |
| --- | --- | --- | --- | --- |
| | 0 nM | 150 nM | 300 nM | 600 nM |
| | 1 | 0.919961 | 1.068998 | 0.950585 |
| | 0.489239 | 0.782649 | 0.9374 | 0.729937 |
| | 1 | 1.234756 | 1.157949 | 0.943369 |
| | 0.309463 | 0.573195 | 0.608046 | 0.498942 |
| average | 0.699676 | 0.87764 | 0.943098 | 0.780708 |
| stdev | 0.354466 | 0.277509 | 0.241042 | 0.213921 |
| ttest | 1 | 0.459252 | 0.299385 | 0.708989 |

## Slide 6
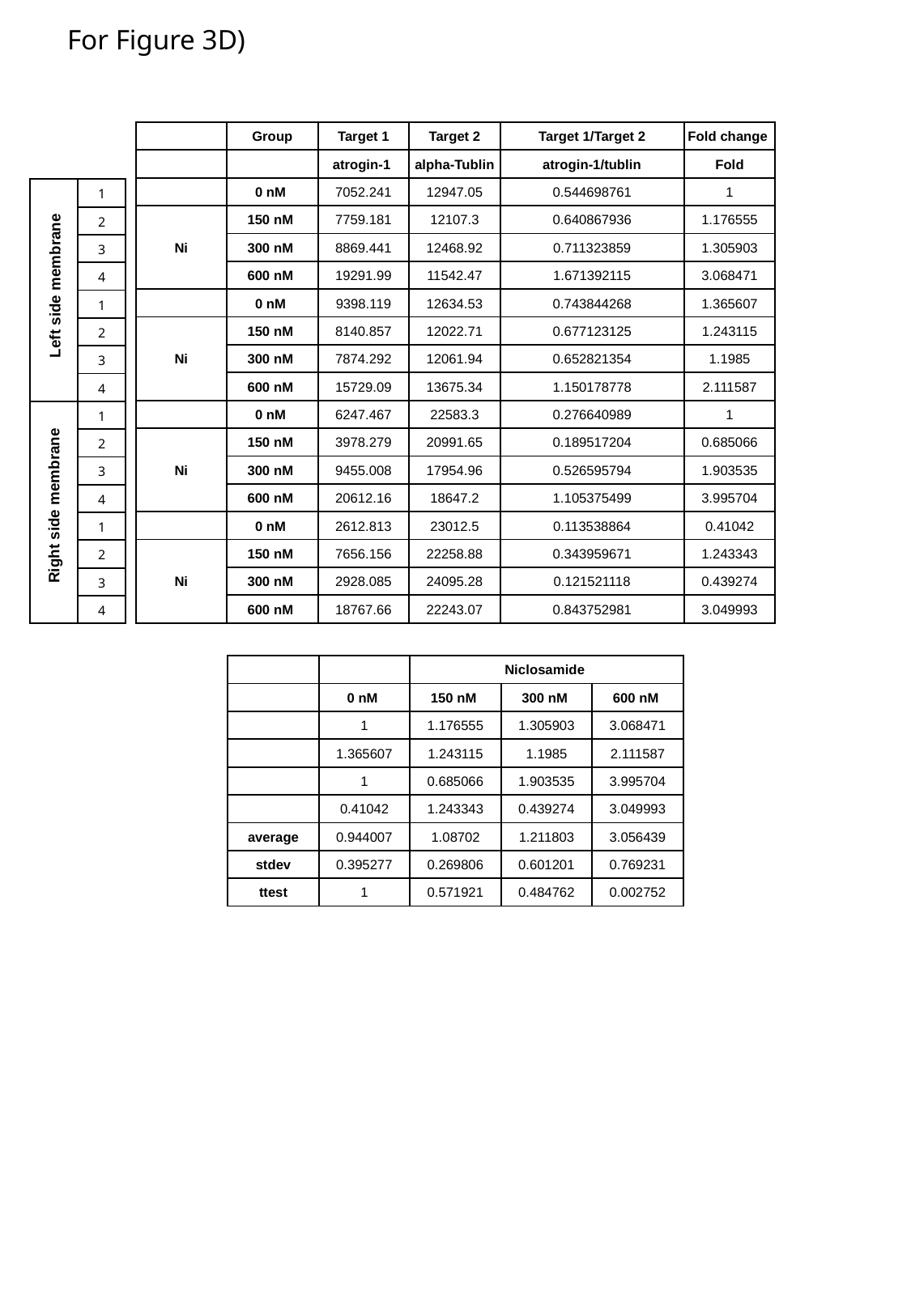

For Figure 3D)
| | Group | Target 1 | Target 2 | Target 1/Target 2 | Fold change |
| --- | --- | --- | --- | --- | --- |
| | | atrogin-1 | alpha-Tublin | atrogin-1/tublin | Fold |
| | 0 nM | 7052.241 | 12947.05 | 0.544698761 | 1 |
| Ni | 150 nM | 7759.181 | 12107.3 | 0.640867936 | 1.176555 |
| | 300 nM | 8869.441 | 12468.92 | 0.711323859 | 1.305903 |
| | 600 nM | 19291.99 | 11542.47 | 1.671392115 | 3.068471 |
| | 0 nM | 9398.119 | 12634.53 | 0.743844268 | 1.365607 |
| Ni | 150 nM | 8140.857 | 12022.71 | 0.677123125 | 1.243115 |
| | 300 nM | 7874.292 | 12061.94 | 0.652821354 | 1.1985 |
| | 600 nM | 15729.09 | 13675.34 | 1.150178778 | 2.111587 |
| | 0 nM | 6247.467 | 22583.3 | 0.276640989 | 1 |
| Ni | 150 nM | 3978.279 | 20991.65 | 0.189517204 | 0.685066 |
| | 300 nM | 9455.008 | 17954.96 | 0.526595794 | 1.903535 |
| | 600 nM | 20612.16 | 18647.2 | 1.105375499 | 3.995704 |
| | 0 nM | 2612.813 | 23012.5 | 0.113538864 | 0.41042 |
| Ni | 150 nM | 7656.156 | 22258.88 | 0.343959671 | 1.243343 |
| | 300 nM | 2928.085 | 24095.28 | 0.121521118 | 0.439274 |
| | 600 nM | 18767.66 | 22243.07 | 0.843752981 | 3.049993 |
| | 1 |
| --- | --- |
| | 2 |
| | 3 |
| | 4 |
| | 1 |
| | 2 |
| | 3 |
| | 4 |
| | 1 |
| | 2 |
| | 3 |
| | 4 |
| | 1 |
| | 2 |
| | 3 |
| | 4 |
Left side membrane
Right side membrane
| | | Niclosamide | | |
| --- | --- | --- | --- | --- |
| | 0 nM | 150 nM | 300 nM | 600 nM |
| | 1 | 1.176555 | 1.305903 | 3.068471 |
| | 1.365607 | 1.243115 | 1.1985 | 2.111587 |
| | 1 | 0.685066 | 1.903535 | 3.995704 |
| | 0.41042 | 1.243343 | 0.439274 | 3.049993 |
| average | 0.944007 | 1.08702 | 1.211803 | 3.056439 |
| stdev | 0.395277 | 0.269806 | 0.601201 | 0.769231 |
| ttest | 1 | 0.571921 | 0.484762 | 0.002752 |

## Slide 7
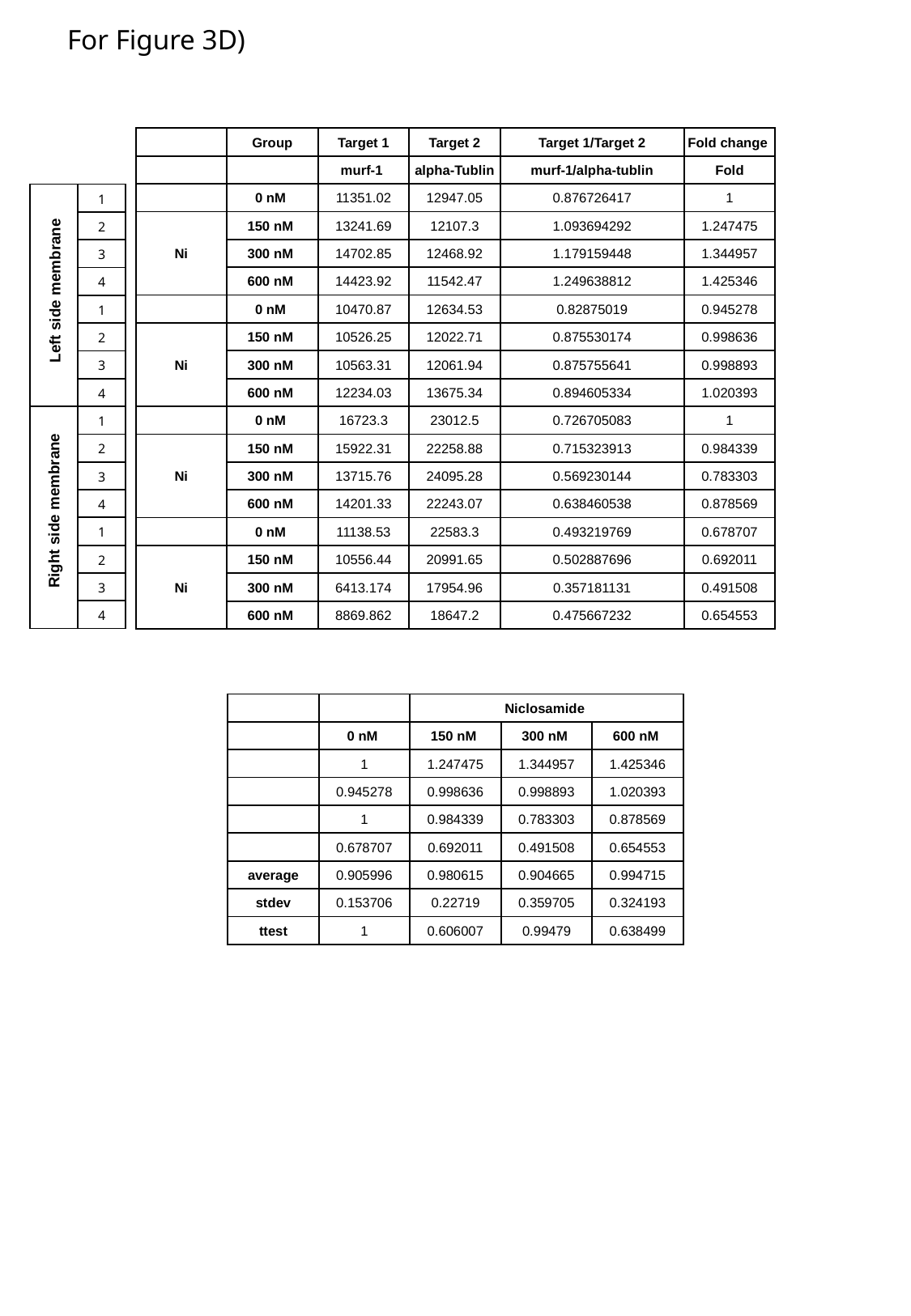

For Figure 3D)
| | Group | Target 1 | Target 2 | Target 1/Target 2 | Fold change |
| --- | --- | --- | --- | --- | --- |
| | | murf-1 | alpha-Tublin | murf-1/alpha-tublin | Fold |
| | 0 nM | 11351.02 | 12947.05 | 0.876726417 | 1 |
| Ni | 150 nM | 13241.69 | 12107.3 | 1.093694292 | 1.247475 |
| | 300 nM | 14702.85 | 12468.92 | 1.179159448 | 1.344957 |
| | 600 nM | 14423.92 | 11542.47 | 1.249638812 | 1.425346 |
| | 0 nM | 10470.87 | 12634.53 | 0.82875019 | 0.945278 |
| Ni | 150 nM | 10526.25 | 12022.71 | 0.875530174 | 0.998636 |
| | 300 nM | 10563.31 | 12061.94 | 0.875755641 | 0.998893 |
| | 600 nM | 12234.03 | 13675.34 | 0.894605334 | 1.020393 |
| | 0 nM | 16723.3 | 23012.5 | 0.726705083 | 1 |
| Ni | 150 nM | 15922.31 | 22258.88 | 0.715323913 | 0.984339 |
| | 300 nM | 13715.76 | 24095.28 | 0.569230144 | 0.783303 |
| | 600 nM | 14201.33 | 22243.07 | 0.638460538 | 0.878569 |
| | 0 nM | 11138.53 | 22583.3 | 0.493219769 | 0.678707 |
| Ni | 150 nM | 10556.44 | 20991.65 | 0.502887696 | 0.692011 |
| | 300 nM | 6413.174 | 17954.96 | 0.357181131 | 0.491508 |
| | 600 nM | 8869.862 | 18647.2 | 0.475667232 | 0.654553 |
| | 1 |
| --- | --- |
| | 2 |
| | 3 |
| | 4 |
| | 1 |
| | 2 |
| | 3 |
| | 4 |
| | 1 |
| | 2 |
| | 3 |
| | 4 |
| | 1 |
| | 2 |
| | 3 |
| | 4 |
Left side membrane
Right side membrane
| | | Niclosamide | | |
| --- | --- | --- | --- | --- |
| | 0 nM | 150 nM | 300 nM | 600 nM |
| | 1 | 1.247475 | 1.344957 | 1.425346 |
| | 0.945278 | 0.998636 | 0.998893 | 1.020393 |
| | 1 | 0.984339 | 0.783303 | 0.878569 |
| | 0.678707 | 0.692011 | 0.491508 | 0.654553 |
| average | 0.905996 | 0.980615 | 0.904665 | 0.994715 |
| stdev | 0.153706 | 0.22719 | 0.359705 | 0.324193 |
| ttest | 1 | 0.606007 | 0.99479 | 0.638499 |
